# Australian Students’ Perceptions of Their Teachers’ Self-Regulated Learning Strategy Instruction

**DOI:** 10.3390/bs15121643

**Published:** 2025-11-29

**Authors:** Carolyn Murdoch, Sean H. K. Kang, Emily White, Lorraine Graham

**Affiliations:** Faculty of Education, The University of Melbourne, Melbourne, VIC 3010, Australia; sean.kang@unimelb.edu.au (S.H.K.K.); emily.white@unimelb.edu.au (E.W.); lorraine.graham@unimelb.edu.au (L.G.)

**Keywords:** self-regulated learning, teacher SRL practices, teacher SRL knowledge, measurement of SRL promotion, student perspective of SRL promotion

## Abstract

While research has established the importance of Self-Regulated Learning (SRL) strategies for student achievement, their effective instruction in classrooms is often lacking. This study adopted a novel methodology that focused on Australian students’ perspectives of their teachers’ promotion of SRL strategies. Eight secondary school teachers completed a professional learning programme aimed at promoting SRL during regular classroom instruction and submitted a video excerpt of their instruction. These videos were used as stimuli for semi-structured stimulated recall interviews conducted with 25 students. Students were asked to describe their teachers’ SRL strategy instruction in terms of ‘What, When, Why and How?’. Associations between instances where students provided a clear description of the purpose and possibilities for transfer of SRL strategies and their teachers’ actions, manner of promotion and choice of strategy type were explored. Results indicate that SRL instruction was most noticed by students when it consisted of naming the strategy, providing a clear process to be followed to apply the strategy, and was accompanied by teachers’ explanations about how and why the strategy improves learning, combined with prompts to encourage students to provide examples of transfer. The implications of these results for how teachers can best promote SRL in the classroom are discussed.

## 1. Introduction

Self-regulated learning (SRL) has been positively associated with increased learning behaviour, motivation and achievement and is generally accepted to be an important skill for academic and lifelong learning (Dignath & Veenman, 2021; Dent & Koenka, 2016; Karlen, 2016). To ensure all students develop these important skills, it is important that their teachers can access and use the knowledge, beliefs and practices necessary to effectively promote SRL in classrooms.

This research describes the instructional practices that promote SRL strategies used by a small sample of Australian secondary school teachers and it builds on the work of two Australian research teams—the Realising the Potential of High Capacity Students Project (REAP), which investigated students’ SRL capabilities (Harding et al., 2018), and the Teaching How to Learn Project, which investigated teacher actions to promote students’ SRL (Stephenson et al., 2024; Vosniadou et al., 2024). The current research focused on authentic secondary school classroom lessons, unlike other recent Australian research, which focused on primary school students (Alvi & Gillies, 2021; Brinkman et al., 2025) or tertiary students (Arianpoor et al., 2025; Higgins et al., 2021). This is particularly important in the Australian setting due to the recent recommendations by education departments across the country for SRL skills to be embedded in classroom instruction at all levels, in order to enhance students’ immediate academic outcomes and lifelong learning (Department of Education Victoria, 2025; New South Wales Department of Education, 2020). By analysing authentic instances of SRL strategy promotion captured through classroom video recordings, this study provides insights into SRL instruction. Importantly, these instances of teachers’ SRL strategy promotion were investigated to collect evidence of the students’ perspectives on strategy promotion by examining their statements regarding ‘what to do, why to do it and when to do it’. These student descriptions serve as indicators of the clarity of SRL strategy promotion, allowing for the identification of teacher actions associated with more accurate student descriptions of the purpose and potential transfer of the SRL strategies to other learning situations. For the purpose of this research, SRL strategies were defined as any process or activity that learners use automatically or deliberately to enhance their learning (Lawson et al., 2019). This definition was adopted as the work of Lawson et al. was foundational to the Teaching How to Learn Research Project, from which this study evolved. Students require a range of SRL strategies, or an “SRL toolkit”, along with an understanding of how, when, and where to use these (Vosniadou et al., 2021).

Effective SRL instruction and promotion has been investigated across various educational contexts, including primary, secondary and tertiary levels (Karlen et al., 2025; Sins et al., 2024; Țepordei et al., 2025) as well as more recently for online and flipped learning environments (Ateş, 2024). Dignath and Büttner (2018) distinguish between direct and indirect SRL promotion in classrooms. Indirect promotion involves creating a learning environment where students can take an active role or responsibility for their learning, whereas direct instruction involves the instruction of learning strategies to be used by the students to manage their learning (Dignath & Büttner, 2018; Dignath & Veenman, 2021). While indirect promotion of SRL is important to allow students to practice and receive feedback on their SRL skills, the current study focused on the direct instruction of SRL strategies in secondary school classrooms.

Direct instruction of SRL strategies can be explicit or implicit; however, definitions of these vary. A key area for investigation in the field concerns the definition and implementation of explicit SRL strategy instruction (Dignath-van Ewijk et al., 2013; Veenman et al., 2006; Vosniadou et al., 2024). While researchers generally agree that explicit instruction involves making students aware of strategy instruction and providing clear guidelines for strategy use, there are variations in the specific components deemed necessary for effective explicit instruction (Dignath-van Ewijk et al., 2013; Veenman et al., 2006; Vosniadou et al., 2024). Veenman et al.’s (2006) ‘WWW&H rule’ emphasises explaining what to do, when, why, and how to use a strategy, while Dignath-van Ewijk et al.’s (2013) definition also includes highlighting the benefits of strategy use. Dignath and Veenman (2021) underscored the importance of explicit instruction in supporting students’ SRL, based on a systematic review of classroom observation studies. However, they also noted that more research is needed to develop effective programmes for enhancing teacher capacity to foster students’ SRL, as current instruction in classrooms remains uneven and inequitable (Greene, 2021).

The need for increased professional learning for teachers targeting SRL knowledge, beliefs and classroom practice has been highlighted by several studies. Harding et al. (2018) found a significant discrepancy between teachers’ beliefs about SRL’s importance and their actual classroom practices. While 98.8% of teachers agreed that SRL was important, only 32% of teachers surveyed indicated that they specifically planned to include aspects of SRL in their lessons. Moreover, a further 23% of the 124 teachers surveyed acknowledged that they did not know how to teach SRL. This gap in knowledge and practice seems particularly pronounced for secondary school teachers, who were found to explicitly instruct and use SRL strategies less than their primary school colleagues. Michalsky (2021) further emphasised this issue, noting that the “apparent deficit in most teachers’ basic SRL teaching pedagogical awareness is striking” (Michalsky, 2021, p. 7) when compared to other pedagogical domains such as classroom management.

Dignath and Büttner’s (2008) meta-analysis of SRL training programmes provided additional insights into the challenges of SRL promotion. Their meta-analysis of 35 secondary school studies found that SRL can be effectively promoted by teachers to improve student strategy use and academic performance; however, a concerning finding was that the impact of SRL training on students’ strategy use was greater when delivered by researchers rather than regular classroom teachers. A more recent finding by Eberhart et al. (2025) in their meta-analysis of young children’s SRL contradicts this, which they suggested may reflect recent improvements in how teachers and researchers collaborate to develop SRL programmes. In addition, this difference may be the result of the young age of the children in the study. However, only classroom teachers are able to embed SRL strategy instruction in the content of the lesson, an effective instructional practice recommended by Hattie and Donoghue (2016) and Michalsky (2021). This highlights the need for more comprehensive SRL knowledge, understanding, and professional learning for classroom teachers to ensure effective delivery of SRL programmes (Heaysman & Kramarski, 2022).

To address these challenges, there is a clear need to understand teachers’ current beliefs about SRL (Lawson et al., 2019; Karlen et al., 2023) and to build their capacity and confidence in teaching these skills. Kramarski (2018) introduced the concept of self-regulated teaching (SRT) and advocated for professional learning programmes that explore teachers’ SRL beliefs and practices, emphasise the importance of SRL for lifelong learning, and build teachers’ capacity to provide embedded, explicit SRL instruction (Kramarski & Heaysman, 2021).

The main aim of this research is to develop a comprehensive picture of effective teacher instructional practices for SRL promotion, focusing on how teachers communicate the ‘what, when, why, and how’ of SRL strategies. Importantly, this study addresses a critical gap in the literature by incorporating students’ perceptions and interpretations of teachers’ classroom practices related to SRL, an area highlighted by Karlen et al. (2025) as under-researched. By designing a methodology to access these student perspectives in authentic classroom contexts, this research contributes to the field. Its goal is to enhance our understanding of effective SRL promotion in secondary school classrooms by examining both teacher practices and student perceptions. The findings will inform the development of targeted professional learning programmes, ultimately contributing to more equitable and effective SRL instruction for all students across educational settings.

This study builds upon the research conducted by the Teaching How to Learn (THtL) Research team, which developed the Self-regulated Learning Teacher Promotion Framework (SRL-TPF) for direct promotion of SRL in classrooms (Vosniadou et al., 2024). The THtL research team used this Framework to create professional learning modules aimed at enhancing teachers’ knowledge of SRL and understanding of explicit SRL strategy instruction (Stephenson et al., 2024).

The current research expands on previous work by incorporating students’ perspectives on their teachers’ SRL instruction practices. This approach allows for the collection of evidence regarding students’ ability to detect, understand, and potentially apply the SRL strategies taught by their teachers. The effective instruction of SRL strategies is expected to enhance students’ perception and understanding of SRL strategies and when and where to use them.

This study was designed to investigate three research questions:

RQ 1: How are teachers promoting SRL in secondary classrooms?
RQ 2: To what extent are the students able to describe the SRL strategy being promoted by their teacher?
RQ 3: Which SRL strategy instruction and promotion types may increase students’ recognition of SRL strategies?

## 2. Materials and Methods

**Figure 1:**
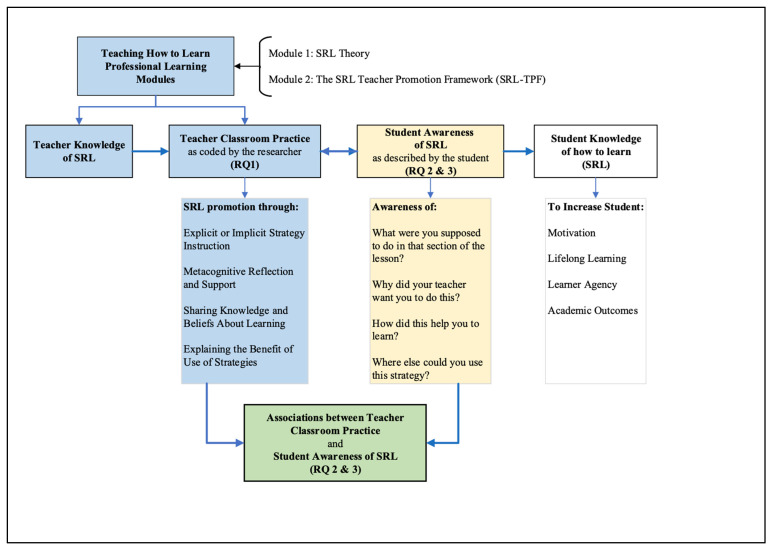
The Research Framework.

To investigate these questions, we employed a multi-faceted approach combining video analysis of classroom instruction, stimulated recall interviews with students, and quantitative analysis of the relationships between teacher instruction and student understanding. Research Question 1 was explored using videos of authentic classroom instruction submitted by each of the eight participating teachers. Instances of SRL strategy instruction were extracted from these videos and each instance of strategy instruction was coded using the categories described in the SRL-TPF to calculate the frequency of instruction of each type of strategy along with the frequency of each category of teacher actions used to instruct each SRL strategy. Research Questions 2 and 3 were explored from the students’ perspective by conducting Stimulated Recall Interviews with students and collecting descriptions of their teachers’ SRL instruction. These descriptions were assessed according to the students’ ability to (1) explain the purpose of the strategy and (2) provide examples of other classroom situations where the strategy could be used and (3) provide examples of other learning situations, outside of a classroom, where the strategy could be used to learn. Research Questions 2 and 3 were investigated using quantitative analysis of the data to establish the strength and direction of associations between the types of strategies (RQ 2), the types of teacher instruction (RQ 3) and the students’ descriptions of each strategy. The relevance of the data collected for each research question is demonstrated in the Research Framework (Figure 1).

### 2.1. Contexts and Participants

Due to COVID-19 pandemic restrictions in 2022 and associated time constraints, data were collected from a small sample of three schools in metropolitan Melbourne, Australia. The sample included two government schools and one independent girls’ school. Eight teachers volunteered to complete the online SRL Professional Learning Modules and participate in the research. These teachers represented a range of teaching experience (from less than 5 years to over 25 years) and disciplinary subjects.

Each teacher selected one of their classes for video recording. A total of 25 students from these eight videoed classes provided written consent and participated in interviews. The participating students were not evenly distributed across classes or year levels (years 7 to 11) due to the voluntary nature of the interviews. Two students completed the interview online, while 23 completed it in person. As this research was funded by the Teaching How to Learn collaborative project between Flinders University and the University of Melbourne, this study was approved by the Social and Behavioural Research Ethics Committees of Flinders University and this approval was registered by the University of Melbourne. All participants provided informed consent before participating in the study.

### 2.2. Research Procedure and Data Collection

The lesson videos submitted by the teachers were analysed to produce data to explore RQ 1, and these same videos were used as a stimulus for a student stimulated recall interview, the data from which was used to explore RQ 2 and 3.

#### 2.2.1. Research Procedure and Data Collection to Investigate Research Question 1

Classroom videos showing each of the participating teachers instructing a class were submitted by the teachers at the completion of the THtL professional learning programme. These videos were edited to show only the instances of SRL strategy instruction identified by the researcher. Each instance of SRL strategy instruction was then analysed and coded by the researcher using the definitions and descriptions provided by the THtL coding guide (Appendix A). This THtL coding guide contained definitions of each of the categories described in the SRL-TPF for direct promotion (Vosniadou et al., 2024, p. 396), along with examples of the type of statements teachers might make when using specific categories of instruction and promotion. The THtL team revised the coding guide until interrater reliability reached substantial agreement (84% with a Cohen’s *k* of 0.68). The coding process involved several steps:

- Categorising instruction as explicit or implicit
- Identifying types of promotion used (metacognitive reflection and support, knowledge and beliefs about learning, or benefit of use)
- Coding the manner of promotion (direct verbal, modelling, or prompting)
- Categorising the purpose of the strategy (cognitive, metacognitive, motivational, or affective)
- Determining the domain of each strategy (domain-general or domain-specific)

This multi-faceted coding of the observed instances of strategy instruction was used to construct a detailed picture of teachers’ classroom practices to investigate Research Question 1.

#### 2.2.2. Research Procedure and Data Collection to Investigate Research Questions 2 and 3

The same edited video segments of SRL strategy instruction that had been coded by the researcher were then used as a stimulus for a stimulated recall interview with students. This methodology allowed students to describe their understanding of their teacher’s SRL instruction practice without interrupting the flow of the lesson or relying on memory.

During the 30 min stimulated recall interviews, students viewed video excerpts of their teacher’s SRL strategy instruction (ranging from six to 10 min in length) and answered questions derived from Veenman et al.’s ‘What to do, When, Why and How Rule’ (Veenman et al., 2006). These questions were verbally stated as well as available as a hard copy:

1. “What were you supposed to do in this section of the lesson?”
2. “What did your teacher do, say, make or write to let you know this was expected?”
3. “Why did your teacher want you to do this?”
4. “How would this help you to learn?”
5. “Where else could you use this learning strategy?”

Each student interview was recorded and transcribed to identify instances where students had identified the purpose of each strategy or provided examples of transfer to other classroom situations (near transfer) or to learning situations outside of a classroom (far transfer). While the five interview questions were used to guide responses, each student’s entire transcript was considered when looking for evidence of statements that indicated that students had understood the purpose of the strategy or could provide examples of transfer.

A stimulated recall interview coding guide (Appendix B) for student responses was developed and refined through multiple trials to ensure reliability. It was first trialled with four researchers and the possible student statements re-defined, then further trialled with two researchers, and the appropriate clarifications made. Once 94% agreement was obtained, one researcher completed the coding of the students’ responses.

The probability that students would accurately describe the purpose of the SRL strategy and provide examples of transfer of the strategy was calculated in order to explore the extent to which students could describe each type of strategy. The odds ratios that these descriptions were associated with the type of strategy were also calculated to explore the strength and direction of these associations. The analysis involved converting each category of strategy type and teacher action to binary data (1 = presence, 0 = absence) and calculating the weighted average probability (due to the uneven number of participating students per given teacher) of students accurately describing the strategy’s purpose and providing examples of near and far transfer. Logistic regressions were conducted using the *Minitabs*^®^ (Version 21) programme (Minitab LLC, 2021), which yielded odds ratios, confidence intervals and p-values for the association of each strategy type and teacher action with each of the three student measures. The resulting probabilities and positive statistically significant odds ratios for the different strategy types were used to answer Research Question 2. The probabilities and odds ratios for the association of teacher actions with the student measures were used to identify which teacher actions were most effective in promoting student understanding and transfer of SRL strategies, to provide an answer to Research Question 3.

## 3. Results

### 3.1. Teachers’ SRL Instruction

To provide a comprehensive overview of the types of SRL strategies and the teacher actions used to instruct and promote these strategies that were apparent to the researcher, we calculated the frequency of each teacher action and strategy type observed. Table 1 presents examples of the strategies instructed by the teachers, categorised by their strategy type.

Cognitive strategies were the most frequently instructed, accounting for 60% (25 out of 42) of all strategies observed. This was nearly twice the frequency of metacognitive strategies, which comprised 31% (13 out of 42) of the total. Due to their infrequent occurrence (9%, four out of 42) and similar nature from a student perspective, motivational and affective strategies were combined to allow for statistical analysis.

The teachers’ actions used to instruct and promote these SRL strategies varied considerably in frequency, with clear patterns emerging. Explicit instruction was twice as prevalent as implicit instruction, with 28 of the 42 strategies (66.6%) explicitly instructed. Notably, 100% (four out of four) of the motivational/affective strategies were explicitly instructed.

Regarding promotion types, metacognitive reflection and support were the most frequently occurring, accompanying 71% of the strategy instruction evident in the sample. Knowledge and beliefs were used to promote 52% (22 out of 42) of the SRL strategies, while the benefit of use of the strategy was only emphasised for 40% (17 out of 42) of the strategies. Teachers most frequently used two promotion types together (36% out of strategies, 15 out of 42) and employed two or three types of promotion in 57% (24 out of 42) of the strategies instructed. Conversely, 14% (six out of 42) of the strategies were instructed without any additional promotion.

In terms of manner of promotion, direct verbal instruction was most frequently used (52%, 22 out of 42), followed by prompting (31%, 13 out of 42) and then modelling (17%, 7 out of 42), as shown in Table 2. This table also includes information about the frequency of the purpose and domain of each strategy, with cognitive strategies being the most frequent (as previously shown in Table 1) and 86% of SRL strategies coded as domain general.

### 3.2. Student Description of the SRL Strategies

To understand which teacher actions may have enabled students to provide descriptions of the purpose of each SRL strategy or provide examples of transfer, we calculated the weighted average probability for each measure. Unexpectedly, the highest probability of students accurately reporting the purpose of the strategy and providing examples of near and far transfer was obtained for strategies that were implicitly instructed.

The teacher actions associated with the highest weighted average probabilities were promotion of the strategy using knowledge and beliefs about learning, using two or more promotion types, and using prompting as the manner of promotion (see Table 2).

Regarding strategy types, cognitive strategies were associated with the highest weighted average probabilities for each of the three student measures. Domain-general strategies were associated with a higher probability of students providing examples of each type of transfer. However, the highest probability of students describing the purpose of the strategy was obtained for domain-specific strategies (see Table 3).

### 3.3. SRL Strategy Instruction Associated with Detailed Student Descriptions

We identified several teacher actions that were significantly positively associated with detailed student descriptions (which included describing the purpose of the strategy and providing examples of near and far transfer). Table 4 lists the odds ratios of the significant associations. These teacher actions were using knowledge and beliefs to promote the SRL strategy and using two or more types of promotion. Conversely, there was a significant negative association between students’ ability to describe both the purpose and provide examples of transfer when teachers used one or fewer types of promotion for the SRL strategy instruction.

The manner of promotion used by the teacher was also associated with both types of transfer. When teachers used prompting to promote the strategy, this was significantly positively associated with the provision of examples of both near and far transfer. In contrast, direct verbal promotion was significantly negatively associated with both types of transfer.

Implicit instruction of strategies was significantly positively associated with instances of students providing examples of near transfer to another classroom learning situation. Conversely, as the binary opposite of this finding, explicit instruction was significantly negatively associated with near transfer.

With regard to strategy types, cognitive strategies were significantly positively associated with students reporting the purpose of the strategy and providing examples of near transfer. Motivational/affective strategies had significant negative associations for both of these measures. Similarly, Metacognitive strategies were significantly negatively associated with students reporting the purpose of the strategy. There were no significant associations between the Domain of the strategy (general or specific) and the three student measures, as shown in Table 4.

## 4. Discussion

This study aimed to investigate how teachers promote SRL in secondary classrooms, the extent to which students can describe SRL strategies that are instructed, and which instruction and promotion types may increase students’ recognition of these SRL strategies. The findings provide insights into effective SRL instruction and have important implications for teacher professional development and classroom practice.

RQ 1: How are teachers promoting SRL in secondary classrooms?

Our analyses revealed that teachers in this study most frequently instructed cognitive, domain general strategies using explicit instruction. They predominantly used a direct verbal manner of promotion and provided metacognitive reflection and support combined with one other promotion type.

The higher frequency of instruction of cognitive strategies compared to metacognitive or motivational/affective strategies aligns with previous research (Dignath & Büttner, 2018; van Loon et al., 2021; Vosniadou et al., 2024). This pattern may be explained by teachers’ perceptions of time constraints in covering curriculum content alongside SRL skills, as reported by Harding et al. (2018). This finding raises concern as it may suggest that teachers perceive the promotion of metacognitive and motivational/affective strategies as secondary to their primary role of transmitting content-specific information (Kramarski & Michalsky, 2009; Lane, 2015; Vosniadou et al., 2021). To address this issue, it is crucial to convince teachers of the importance of instructing SRL strategies across all domains and provide them with clear instructional models to ensure effective, planned instruction.

The prevalence of direct verbal instruction observed in the videos submitted may be related to the data collection process as teachers likely videoed examples of whole-class instruction at the start of lessons or when introducing new SRL strategies. In both situations, it would be expected that teachers would provide more verbal instruction to their students. This finding aligns with Vosniadou et al.’s (2024) study which reported similar frequencies of direct verbal instruction and promotion of domain general strategies. However, the increased frequency of explicit instruction in our study diverges from Vosniadou et al.’s findings, possibly due to the influence of the THtL professional learning modules completed by the teachers in our study. These modules emphasised the explicit instruction of SRL strategies, encouraging teachers to name strategies and clearly explain their processes. The completion of these modules may also explain the higher frequency of teachers incorporating knowledge and beliefs about learning and metacognitive reflection coded in our research compared to Vosniadou et al.’s (2024) study. This would suggest that the participating teachers were able to integrate key messages from the THtL modules into their recorded lessons. Other professional learning programmes have produced similar increases in teachers’ use of examples of explicit instruction (Sins et al., 2024).

RQ 2: To what extent are the students able to describe the SRL strategy being promoted by their teacher?

Our analysis of students’ responses to the stimulated recall interview questions revealed that cognitive strategies were associated with the most detailed student descriptions. These strategies were the only type positively associated with students’ ability to describe the purpose and transfer of the strategies. While no associations were found between the domain (general or specific) of the strategy and the students’ descriptions, students struggled to provide examples of transfer for domain-specific strategies. This would be expected due to the subject-specific nature of these strategies, highlighting the need for teachers to clearly communicate real-world applications for domain-specific strategies. The strong positive association between cognitive strategies and student descriptions of their purpose (OR = 4.847) reflects the high probability (83.3%) of students reporting these strategies. Students were nearly five times more likely to report the purpose of cognitive strategies than they were for metacognitive or motivational/affective strategies. This finding may indicate that students perceive cognitive strategies as more valuable and memorable, aligning with Cartier et al.’s (2010) observation that students primarily focus on subject content and cognitive strategies, drawing connections between their use and learning outcomes. This is supported by more recent findings that students most frequently reported cognitive strategies such as rehearsal (Olop et al., 2024) and considered this strategy to be most effective for learning (Kikas et al., 2022). These results also demonstrate that students can accurately describe SRL strategies that are evident to them and that they value, highlighting the need for teachers to ensure that metacognitive and motivational/affective strategies are also promoted effectively and the value or benefit of learning them is clearly communicated to students.

Students were least likely to provide detailed descriptions for motivational/affective strategies, with negative associations reported for accurately describing their purpose (OR = 0.320) and providing examples of near transfer (OR = 0.337). These results suggest that students may not recognise these as learning strategies or believe that managing their thoughts, feelings, emotions and motivation to learn is something that they should implicitly know how to do. However, since not all students have the opportunity to develop these skills at home or in other classes, this creates an equity issue for students who do not have this understanding. Greene (2021) refers to this circumstance as the “hidden curriculum” of SRL and suggests that some students face significant challenges when schools and teachers prioritise content instruction over explicit SRL instruction.

The importance of motivational/affective strategies has also been highlighted by research regarding student motivation during, for example, remote learning during COVID-19 school closures. Students reported feeling that online learning reduced their motivation to learn due to missing their friends (Longmuir et al., 2021), suggesting the need for students to have more strategies for motivating themselves rather than relying on sources of external motivation. However, in a separate study, most students reported that their cognitive understanding during the remote learning period did not suffer (Maher, 2021), which again supports that all students need to develop a repertoire of cognitive strategies for learning. It could be that teachers need to instruct motivational and affective strategies; ‘selling’ the use of these strategies to manage emotions and motivation as an important part of learning. To enhance the effectiveness of this strategy instruction further, it should be embedded (Hattie & Donoghue, 2016) within usual lessons and not separated into separate study skills or wellbeing sessions (Simón-Grábalos et al., 2025).

The lack of detailed student descriptions of the purpose of metacognitive strategies was shown by the low weighted probability of 55.2% that students would accurately report the purpose of these strategies, and the negative association indicated by the odds ratio of 0.368. This is concerning given the established relationship between metacognitive strategies and student performance and achievement (Dent & Koenka, 2016; Ishak et al., 2025; Zepeda et al., 2019). Metacognitive strategies allow students to know themselves as learners, take responsibility for their learning processes, and select the most appropriate strategies to match learning tasks (Anthonysamy, 2021; Peel, 2020). These strategies build the metacognitive knowledge and metacognitive skills that are necessary for students to be able to interact with learning tasks effectively (Biwer et al., 2023; Theobald, 2021) as outlined by Efklides’ (2011) Metacognitive and Affective Model of Self-Regulated Learning (MASRL Model). Therefore, this finding suggests that students may not be aware of when or how to use these important strategies in their independent learning.

Students mentioned during the interviews that five of the instances of strategy instruction (12%) were learning ‘routines’ that were regularly used in their classrooms. The weighted average probability that students could accurately report the purpose of these routines was 68%, suggesting that despite their frequency of use, not all students could describe the purpose of these routine SRL strategies. Possible explanations for this reduced probability could be that, despite previous teacher explanations of the purpose of these strategies, the students may have forgotten or become habituated to their use over time and are unable to describe the strategies in detail. It is also possible that different teachers may use the same strategies for different purposes, causing confusion for students. Therefore, teachers may need to periodically revisit the instruction of these kinds of routine strategies to ensure students understand the use of each strategy, its purpose and potential for transfer.

RQ 3: Which SRL strategy instruction and promotion types may increase students’ recognition of SRL strategies?

Our study identified several key teacher actions that were associated with increased student recognition and description of SRL strategies. These findings provide valuable insights into effective SRL instruction and have implications for teacher professional development and classroom practice.

One of the most significant findings was the importance of teachers including information about their own knowledge and beliefs about learning when promoting SRL strategies. This approach was associated with the highest probabilities of students correctly describing the strategy’s purpose (81.4%), providing examples of near transfer (83%), and far transfer (47.4%). Knowledge and beliefs about learning were the only promotion type significantly associated with all three student outcome measures: accurate reporting of the strategy’s purpose (OR = 3.20), provision of near transfer examples (OR = 2.90), and far transfer examples (OR = 2.62). While this promotion type was rarely used in isolation, making it difficult to draw firm conclusions about its independent effectiveness, the strong associations suggest that it is a critical element in SRL strategy instruction, either on its own or in combination with other promotion types.

The provision of metacognitive reflection and support by teachers was also found to be associated with students’ ability to provide examples of strategy transfer. Students were more than twice as likely to provide examples of both near and far transfer when this promotion type was present (OR = 2.403 and 2.540, respectively). Interestingly, while there was a high probability (71%) of students accurately reporting the strategy’s purpose when promoted using metacognitive reflection and support, this association was not statistically significant.

The manner of promotion, particularly prompting, emerged as a significant factor in enhancing students’ ability to provide examples of strategy transfer. Students were five times more likely to provide examples of near transfer (OR = 5.729) and more than twice as likely to provide examples of far transfer (OR = 2.324) when prompting was used compared to direct verbal instruction or modelling. This finding may be related to the close connection between prompting and metacognitive reflection and support, as teachers often use questioning to encourage students to reflect on their existing knowledge.

Contrary to previous research (Dignath & Büttner, 2018), our study found no significant association between teachers’ promotion of the benefit of strategy use and students’ provision of detailed descriptions of the strategy’s purpose or transfer. However, we did find a significant association between the use of two or more promotion types and all three student outcome measures. When teachers used two or more promotion types, students were three times more likely to accurately describe the strategy’s purpose (OR = 3.020) and significantly more likely to provide examples of near and far transfer (OR = 2.649 and 2.095, respectively). This finding suggests that the level of detail in teachers’ instruction and promotion increases the likelihood of students noticing, describing, and transferring the target SRL strategy. However, it is important to note that using all three promotion types did not significantly predict any of the student measures, indicating that two types of promotion may be sufficient and that excessive information delivered through direct verbal instruction might lead to students disengaging and tuning out. This finding supports the idea of an optimal level of instructional support in SRL promotion (Dignath & Büttner, 2018; Heaysman & Kramarski, 2022; Kirschner & van Merriënboer, 2017).

In this study, it seems that by using two forms of promotion to highlight the significance of the strategy, particularly knowledge and beliefs about learning and metacognitive reflection and support, teachers maximised the probability that their students would detect the SRL strategy instructed, understand the purpose of the strategy, and be able to provide examples of transfer of the strategy to learning situations both in and out of the classroom. As stated by Dignath and Büttner (2018),

If students are both induced to employ a certain strategy and provided with explicit information about the significance of that strategy, the result should be an improvement in performance and the development of the ability to employ the strategy again when faced with a similar problem.

(p. 131)

Despite previous research focusing on the need for explicit strategy instruction of SRL strategies (Dignath & Büttner, 2018; Michalsky, 2021; Vosniadou et al., 2024), our study found that explicit instruction, as defined in our methodology, was not positively associated with any of the three student outcome measures. Surprisingly, it was negatively associated with students providing examples of near transfer (OR = 0.280). In fact, students were three times more likely to provide examples of near transfer for SRL strategies that had been implicitly instructed (OR = 3.570). This unexpected finding may be attributed to the definition of explicit instruction used in our study, which required the teacher to name the strategy and explain its use in sufficient detail for most students to follow. This definition differs from more comprehensive conceptualisations of explicit instruction in previous research. For instance, Dignath and Büttner (2018), using the Assessing how Teachers Enhance Self-Regulated Learning (ATES) coding system (Dignath-van Ewijk et al., 2013; Dignath & Veenman, 2021), defined explicit instruction as including not only naming and explaining the strategy but also providing information about its benefits and modelling or prompting metacognitive reflection focused on the strategy. This more detailed definition encompasses two types of promotion analysed in our study: benefit of use and metacognitive reflection and support. Our findings show that when two or more promotion types were added to the naming and explanation of an SRL strategy, there was a significant association with all three student description measures. This suggests that what we have uncovered as effective instruction aligns with the ATES coding system (Dignath-van Ewijk et al., 2013; Dignath & Veenman, 2021; Rosenthal et al., 2024).

The ATES coding system was also used by Kistner et al. (2010) in research that found explicit instruction was positively related to student learning gains, which supports our finding that using two or more promotion types leads to more detailed student descriptions of purpose and transfer. Specifically, our results indicate that including information regarding knowledge and beliefs about how learning happens is more effective in building student awareness of strategies than stating the benefit of use for the strategy. These findings advance Veenman et al.’s (2006) WWW& H definition of explicit instruction by providing recommendations to teachers on how to effectively explain these features of each SRL strategy: by using their own knowledge and beliefs about learning, combined with metacognitive reflection and support, and using prompting to encourage students to draw on their existing knowledge and generate their own examples of near and far transfer.

Limitations of this research were created by the small sample size as a result of the COVID-19 pandemic restrictions and disrupted learning experienced during the data collection period in 2022; however, what may be regarded as a limitation is also a key feature of this research, as remote learning experiences may have honed students’ perceptions of their important role in the learning process and highlighted the skills they need to drive their individual learning. Also, this study only captured whole-class instruction and did not capture students’ perceptions of any one-on-one, spontaneous SRL instruction delivered to address individual needs. Finally, it must be acknowledged that the lesson videos analysed were recorded and submitted by the teachers themselves, and this may have resulted in teachers “performing” what they perceived to be expected SRL instruction, instead of their usual instruction; however, the students’ perceptions of this SRL strategy instruction are still useful to unpack and understand.

To further validate and extend these findings, future research with a larger sample size is needed. Collaborative research designs involving teachers would also allow for the investigation of intentional, planned instruction, an aspect not explored in the current study. It is possible that some of the instances of strategy instruction we studied were not planned as SRL instruction, resulting in less comprehensive strategy information being provided. By working closely with teachers to design strategy instruction that systematically controls and measures specific promotion types and their combinations, we could gain deeper insights into effective SRL promotion. Additionally, future studies could build upon the methodology developed in this research to assess student perspectives on their teachers’ SRL strategy instruction. This approach leverages students’ unique position as “critics and creators of educational practice” (Cook-Sather, 2020, p. 182), potentially enhancing both the research outcomes and students’ own learning experiences. Such research directions could significantly contribute to our understanding of effective SRL instruction and inform the development of more targeted and impactful teacher training programmes and classroom practices.

## 5. Conclusions

By adding the students’ perspective, a model of effective SRL strategy instruction can be described. SRL strategy instruction and promotion were most apparent to students when they included two forms of promotion, and most specifically, it included the teacher promoting the strategy through their own knowledge and beliefs about learning and by using metacognitive prompts. In order to build students’ metacognitive strategy awareness, teachers need to convince students of the need to develop their understanding and use of these strategies, since the focus for both teachers and students appears to be on cognitive strategy development. Professional learning for teachers to increase their awareness of ‘what, when, why and how’ SRL strategies should be instructed and used may increase their ability to effectively promote a greater range of strategies to their students.

## Figures and Tables

**Table 1:** SRL Strategies Instructed by Teachers to Build Student SRL Capabilities.

Cognitive Strategies (25)	Metacognitive Strategies (13)	Motivational/Affective Strategies (4)
Highlighting key words (3) Connecting to previous experience (3) Sharing ideas (3) Notetaking (3) Rehearsal (2) Oral Presentation (2) Acronyms (2) Using step-by-step equations (2) Brainstorming (1) Annotating writing/ideas (1) Build a model (1) Summary sheets (1)	Goal setting (4) Activating prior knowledge (3) Copy learning intention and success criteria (3) Checking work (2) Rate understanding (1) Using rubrics (1)	Using rewards (2) Silent writing (1) Word count goal (1)

*Note:* The numbers in brackets indicate the number of instances of this strategy.

**Table 2:** Frequency of Teacher Actions and Types of Strategies.

Teach Action/Strategy Type	Frequency Used by Teachers (*n* = 42)
**Teacher Action**	
**Initial instruction**	
Explicit	28 (66.6%)
Implicit	14 (33.3%)
**Promotion Types**	
Metacognitive reflection and support	30 (71%)
Knowledge and beliefs about learning	22 (52%)
Benefit of use	17 (40%)
**Number of Promotion Types**	
3 promotions	9 (21%)
2&3 Promotions	24 (57%)
0&1 Promotions	18 (43%)
0 Promotions	6 (14%)
**Manner of Promotion**	
Direct verbal	22 (52%)
Prompting	13 (31%)
Modelling	7 (17%)
**Type of Strategy**	
**Purpose of Strategy**	
Cognitive	25 (60%)
Metacognitive	13 (31%)
Motivational/affective	4 (9%)
**Domain of Strategy**	
General	36 (86%)
Specific	6 (14%)

*Note.* More than one promotion type can co-occur in each of the 42 instances of strategy instruction. Therefore, the sum of instances of promotion types = 69.

**Table 3:** Weighted Average Probability of Student Measures as a Function of Teacher Action and Strategy Type.

		Student Measures Weighted Average Probability		
	Frequency Used by Teachers (*n* = 42)	Accurate Reporting of Purpose (%)	Near Transfer (%)	Far Transfer (%)
**Teacher action**				
**Initial instruction**				
Explicit	28 (66.6%)	64.6	62.2	32.9
Implicit	14 (33.3%)	72.7	85.4	38.2
**Promotion types**				
Metacognitive reflection and support	30 (71%)	71.7	76.8	40.4
Knowledge and beliefs about learning	22 (52%)	81.4	83.0	47.4
Benefit of use	17 (40%)	73.3	75.5	37.8
**Number of promotion types**				
2 and 3 promotions	24 (57%)	80.0	81.5	47.7
0 and 1 promotions	18 (43%)	56.9	62.5	23.6
0 promotions	6 (14%)	40.9	40.9	18.2
**Manner of promotion**				
Direct verbal	22 (52%)	64.1	57.8	23.4
Prompting	13 (31%)	73.1	89.3	46.4
Modelling	7 (17%)	70.6	64.7	41.2
**Type of strategy**				
**Purpose of strategy**				
Cognitive	25 (60%)	83.3	79.2	38.9
Metacognitive	13 (31%)	55.2	68.1	31.9
Motivational/affective	4 (9%)	44.4	50	27.8
**Domain of strategy**				
General	36 (86%)	67.5	72.2	36.5
Specific	6 (14%)	72.7	63.6	18.2

**Table 4:** Summary of the Odds Ratios for Significant Associations.

	Statistically Significant Odds Ratios		
	Accurate Describing of the Purpose of the Strategy ^a^	Near Transfer ^b^	Far Transfer ^c^
**Teacher action**			
**Initial instruction**			
Explicit	ns	0.280	ns
Implicit	ns	3.570	ns
**Promotion types**			
Knowledge and beliefs about learning	3.200	2.900	2.620
Metacognitive reflection and support	ns	2.403	2.540
Benefit of use	ns	ns	ns
**Number of additional promotions**			
2 and 3 promotions	3.020	2.649	2.095
0 and 1 promotions	0.331	0.378	0.339
0 promotions	0.255	0.202	ns
**Manner of promotion**			
Prompting	ns	5.729	2.324
Direct verbal	ns	0.270	0.371
Modelling	ns	ns	ns
**Strategy type**			
**Purpose of strategy**			
Cognitive	4.847	2.225	ns
Metacognitive	0.368	ns	ns
Motivational/affective	0.320	0.337	ns
**Domain of strategy**			
General	ns	ns	ns
Specific	ns	ns	ns

*Note.* Total of 42 instances of strategy instruction. ns = not a significant predictor. ^a^ The student identified that the purpose of the strategy is either cognitive, metacognitive, or motivational/affective. ^b^ The student provided examples of how to use the strategy in another classroom or school situation. ^c^ The student provided examples of how to use the strategy in another learning situation not at school.

**Figure A1:**
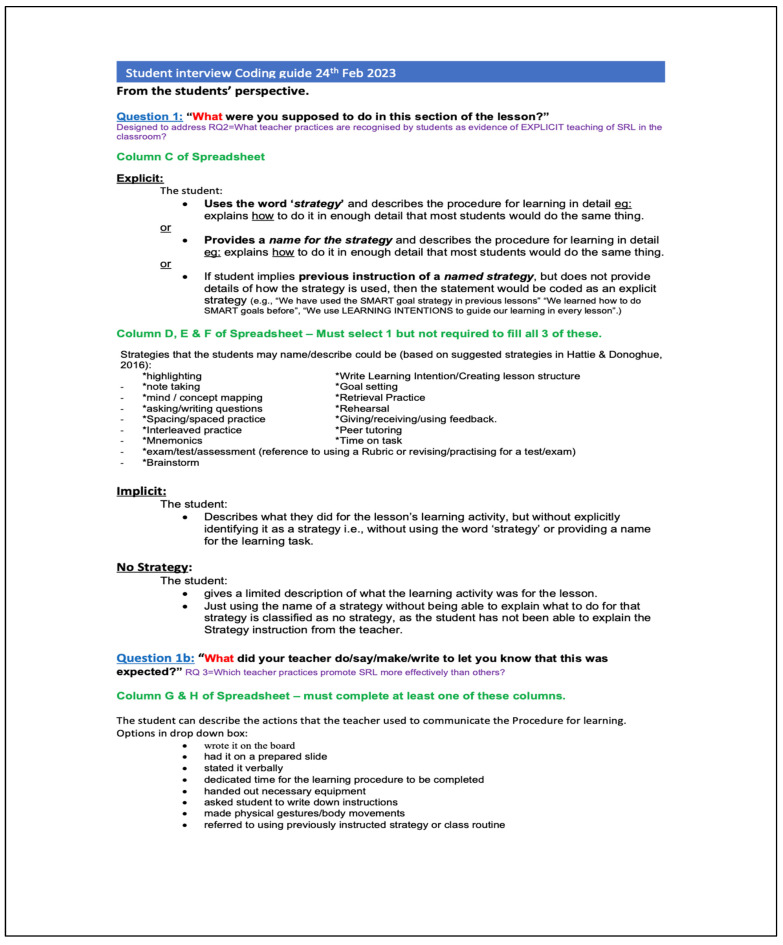

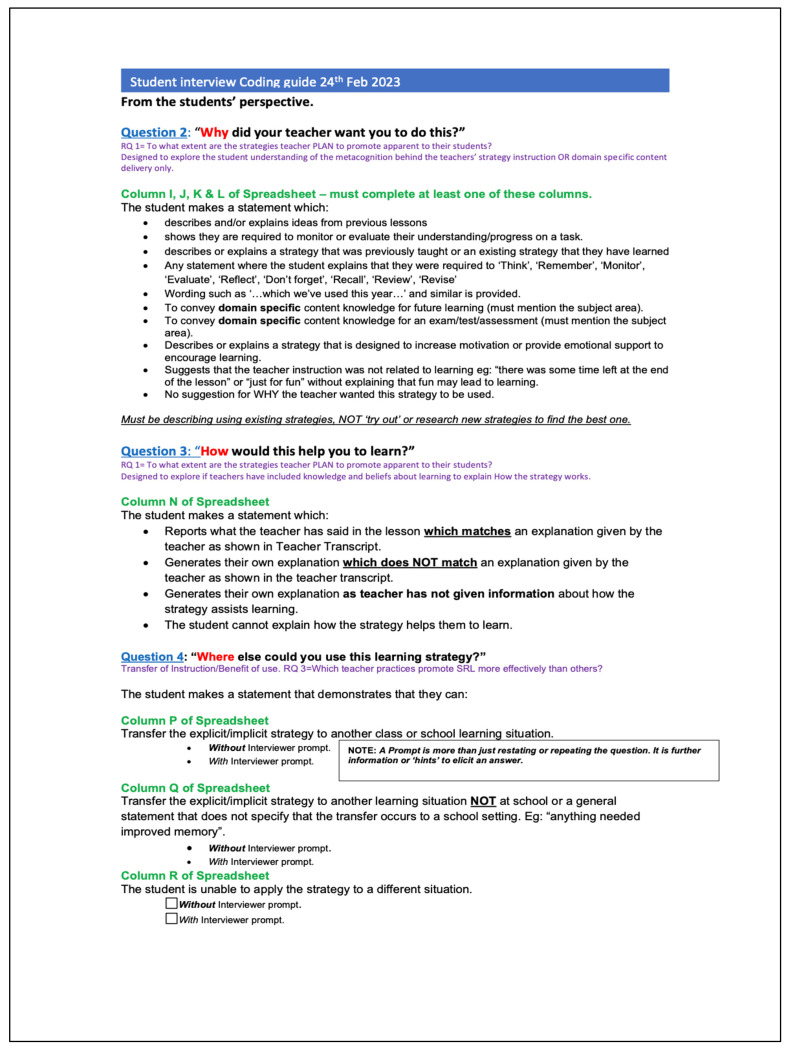
Student interview coding guide.

## Data Availability

Data cannot be shared openly but are available on request from the authors.

## Abbreviations

The following abbreviations are used in this manuscript:

THtL	Teaching How to Learn
SRL	Self-regulated learning
SRL-TPF	Self-regulated Learning Teacher Promotion Framework
WWW&H	What to do, When, Why and How Rule

## Appendix A

**Table A1:** The Self-regulated Learning Teacher Promotion Framework for Direct Promotion.

SRL Promotion Types	SRL Strategy Types	Manner of Promotion	Benefit of Use	Domain
Explicit Instruction: a strategy taught using the word ‘strategy’ or naming the strategy.	Cognitive: strategy used to acquire, organise or use information.	Direct Verbal: teacher tells the students something directly.	Benefit of strategy to support students’ understanding of subject content.	General: related to several subject areas.
Implicit Instruction: a strategy taught without using the word strategy or naming it.	Metacognitive: strategy used to plan, monitor or evaluate learning.	Modelling: teacher shows students how to do something (often with verbal instructions as well)	Benefit of strategy in terms of learning gains.	Specific: related to a specific subject area.
Knowledge and Beliefs about learning: Information is provided about the nature or processes of learning and/or about personal beliefs and their impact on academic performance.	Motivational: strategy used to manage motivation to achieve a goal.	Prompting: teacher provides a hint, cue or encouragement, or asks a question.	Transfer to other tasks where the student could apply the knowledge or tasks.	
Metacognitive Reflection: students are asked to reflect on, monitor or evaluate their own learning.	Affective: strategy used to manage feelings and emotions and their impact on learning			
Metacognitive Support: students are asked to remember and use relevant information or learning strategies.				

*Note.* Adapted from Vosniadou et al. (2024).
